# Transcriptomic and sugar metabolic analysis reveals molecular mechanisms of peach gummosis in response to *Neofusicoccum parvum* infection

**DOI:** 10.3389/fpls.2024.1478055

**Published:** 2024-10-11

**Authors:** Yang Zhang, Yong Liu, Zhimeng Gan, Wei Du, Xiaoyan Ai, Wei Zhu, Huiliang Wang, Furong Wang, Linzhong Gong, Huaping He

**Affiliations:** Hubei Key Laboratory of Germplasm Innovation and Utilization of Fruit Trees, Institute of Fruit and Tea, Hubei Academy of Agricultural Science, Wuhan, China

**Keywords:** peach, gummosis, *N. parvum*, transcriptome, sugar metabolome

## Abstract

Peach gummosis, a devastating disease caused by *Neofusicoccum parvum*, significantly shortens peach tree lifespan and reduces the yield of peach trees. Despite its impact, the molecular mechanism underlying this disease remains largely unexplored. In this study, we used RNA-seq, sugar metabolism measurements, and an integrated transcriptional and metabolomic analysis to uncover the molecular events driving peach gummosis. Our results revealed that *N. parvum* infection drastically altered the transcripts of cell wall degradation-related genes, the log_2_Fold change in the transcript level of *Prupe.1G088900* encoding xyloglucan endotransglycosylase decreased 2.6-fold, while *Prupe.6G075100* encoding expansin increased by 2.58-fold at 12 hpi under *N. parvum* stress. Additionally, sugar content analysis revealed an increase in maltose, sucrose, L-rhamnose, and inositol levels in the early stages of infection, while D-galactose, D-glucose, D-fructose consistently declined as gummosis progressed. Key genes related to cell wall degradation and starch degradation, as well as UDP-sugar biosynthesis, were significantly upregulated in response to *N. parvum*. These findings suggest that *N. parvum* manipulates cell wall degradation and UDP-sugar-related genes to invade peach shoot cells, ultimately triggering gum secretion. Furthermore, weighted gene co-expression network analysis (WGCNA) identified two transcription factors, *ERF027* and *bZIP9*, as central regulators in the downregulated and upregulated modules, respectively. Overall, this study enhances our understanding of the physiological and molecular responses of peach trees to *N. parvum* infection and provide valuable insights into the mechanisms of peach defense against biotic stresses.

## 1 Introduction

Gummosis is characterized by gum exudation from shoots, caused by two main factors: 1) biotic stress by insect and/or fungal attacks, and 2) abiotic stress by mechanical wound and/or environmental factors (Zhang et al., 2022). Most cultivated peach trees are vulnerable to gummosis caused by fungal infection, particularly *Botryosphaeria dothidea*, *Botryosphaeria obtusa*, *Lasiodiplodia theobromae*, and *Neofusicoccum parvum* (Weaver, 1974; Britton and Hendrix, 1982; Beckman and Reilly, 2005; Wang et al., 2011; Gao et al., 2019). These infections lead to severe gum flux, reduced tree vigor, compromised fruit quality, and lower yields (Ezra et al., 2017; Zhang et al., 2023), posing a significant economic threat to the peach industry worldwide, especially in China, Japan, and the United States (Zhang et al., 2022). Some peach varieties, such as ‘Da Hongpao’, ‘Nan Shan Tian Tao’ and ‘Sunfre’, have shown resistance to gummosis, making them potential candidates for breeding programs aimed at developing gummosis-resistant cultivars. However, conventional breeding programs face limitations, including issues with self-compatibility and long juvenile periods (Li et al., 2022; Zhang et al., 2023). Genetic engineering provides an effective strategy to address these challenges. To leverage this approach, it is essential to understand the molecular mechanisms of peach gummosis and identify the genes involved in this process.

Previous research has provided insights into the molecular mechanisms underlying gummosis, revealing the pathological changes in infected peach tissues. Biggs (1988) first demonstrated that parenchyma cells near the periderm and vascular cambium undergo degradation when inoculated with *B. dothidea* and *B. obtusa.* Subsequent research revealed similar cell wall degradation and upregulated expression of cell wall degradation-related genes in response to *L. theobromae* infection (Li et al., 2014a). Gao et al. (2016) identified differentially expressed genes involved in glucometabolic and plant defense response pathways as key players in *L. theobromae*-induced peach gummosis. Zhang et al. (2020) showed that peach trees regulate ROS scavenging enzymes and corresponding genes to suppress *L. theobromae* proliferation. Li et al. (2022) conducted a genome-wide association study (GWAS) and a comparative transcriptome analysis on 195 peach germplasm resources to detect the genetic basis of gummosis resistance. They found five SNPs and six candidate genes, including *galactose oxidase*, an *LRR-RLK*, and an *H2A.1-like*, that are significantly associated with resistance to gummosis. Additionally, Zhang et al. (2023) discovered that ethylene acts as a negative regulator, exacerbating the severity of *L. theobromae* infection. While these studies predominantly focus on peach gummosis caused by *B. dothidea*, *B.obtusa* and *L. theobromae*, *N. parvum*- a pathogen that affects a wide range of hosts-can cause severe diseases in various fruit trees, such as walnut wilt disease and grape leaf spot disease (Cheon et al., 2013; Wu et al., 2015). Despite its wide host range and destructive potential, the molecular mechanism underlying peach’s defense against *N. parvum* remain largely unknown. Sugars play a vital role in plant growth and development, serving as both structural components and major energy sources (Dahro et al., 2016; Xu et al., 2022; Singh et al., 2015; Kretschmer et al., 2017; Khanna et al., 2023). Pathogen attacks disrupt sugar metabolism, leading to the accumulation of specific sugars and polyols (Breia et al., 2020; Zulfiqar et al., 2020; Chen et al., 2024). These sugars can act as signaling molecules, influencing plant hormones, flavonoid synthesis, and ROS production, ultimately contributing to the immune response (Couée et al., 2006; Ferri et al., 2011; Singh et al., 2011). Notably, peach gum is a heteropolysaccharide, suggesting a potential link to cellular sugar metabolism (Khanna et al., 2023). An earlier study has speculated that the peach gum is formed by the degradation of cell wall due to the monosaccharide components of the cell wall are similar to peach gum polysaccharides (Biggs, 1988). While previous studies have shown the disappearance of starch grains and alterations in soluble sugar content upon *L. theobromae* infection (Li et al., 2014a, b), the precise regulatory genes and sugar metabolite changes in the peach-*N. parvum* interaction remain unclear, highlighting the need for further investigation.

As we explore the complex interactions between peach trees and fungal pathogens, a deeper understanding of sugar metabolism and its role in gummosis becomes crucial. This study aims to bridge the knowledge gap by investigating the response of gummosis-susceptible ‘Spring Snow’ peach shoots to *N. parvum* infection over a 60-hour period. Through an integrated analysis of sugar content, metabolomics, and transcriptome sequencing, we aim to identify key stress-responsive genes and elucidate the dynamic changes in sugar accumulation and corresponding biosynthetic pathways. This comprehensive approach will provide a deeper understanding of the molecular mechanisms underlying peach tree responses to *N. parvum*-mediated stress and pave the way for the development of novel gummosis-resistant peach varieties.

## 2 Materials and methods

### 2.1 Plant materials and pathogen

The gummosis-susceptible peach variety ‘Spring snow’ was grafted onto wild peach rootstocks and maintained at the experimental orchard of the institute of Fruit and Tea, Hubei Academy of Agricultural Sciences (Wuhan, China). Current-year shoots were used for the experiments. The *N. parvum* strain JSZ01 was deposited in the Hubei Key Laboratory of Germplasm Innovation and Utilization of Fruit Trees (Wuhan, China). Prior to infection, JSZ01 was cultured on potato dextrose agar (PDA) medium at 26°C for 3 d.

### 2.2 Inoculation of peach shoots with *N. parvum*

The inoculation method followed the (Zhang et al., 2022) with minor modifications. Current-year shoots were defoliated and gently rinsed with flowing water to remove surface dust. The shoots were then cut into approximately 15 cm segments, sterilized with 75% alcohol for 30 s, and washed three times with sterile water. The shoot segments were wounded with a sterile needle and inoculated with *N. parvum* strain JSZ01. As a control, shoots were inoculation with PDA medium. Both treated shoots were placed in a clean plastic box with wet gauzes at the bottom, covered with plastic wrap, and transferred to a light incubator (12 h light/12 h dark, 75% relative humidity) at 26 °C. After inoculation, tissues within 2 cm of the lesion were collected at 12, 24, 36, 48 and 60 h. Each treatment had three replicates, with each replicate consisting of 15 shoot segments. All samples were immediately frozen in liquid nitrogen and stored at -80°C for further analyses.

### 2.3 RNA extraction, cDNA library construction, and RNA-seq

Total RNA from peach shoots was isolated using the Plant Total RNA Kit (Simgen, 5103050, Hangzhou, China) according to the manufacturer’s protocols. The RNA samples were then sent to Biomarker Technologies company (Beijing, China) for paired-end RNA sequencing. The cDNA library was constructed using the NEBNext^®^ Ultra™ RNA Library Prep Kit (NEB, USA) and sequenced using the Illumina Novaseq6000.

To obtain high-quality reads, raw reads were filtered using the default parameters of the fastp software to remove adapter sequences and low-quality reads (Chen et al., 2018). Clean reads were then mapped to the reference genome of *Prunus persica* v2.0 (Verde et al., 2017) using HISAT2. Gene expression levels were calculated using the FPKM method by Feature Counts and R software.

Differential expression of genes (DEGs) between different samples was screened by using DESeq2, with |log2fold changes| ≥ 1 and false discovery ratios (FDR) < 0.05 as the threshold. The DEGs were then subjected to Gene Ontology (GO) and Kyoto Encyclopedia of Genes and Genomes (KEGG) analysis (Kanehisa et al., 2008; Young et al., 2010). GO and KEGG pathways with a q value < 0.05 were considered significantly enriched. The visualization of the heatmap and Bubble Chart was drawn using R language (Gu, 2022; Villanueva and Chen, 2019).

### 2.4 Quantitative real-time PCR

First-strand cDNA was synthesized using a commercial kit (Simgen, 7306100, Hangzhou, China) according to the manufacturer’s instructions. qPCR was performed on the QuantStudio 7 Flex instrument (Applied Biosystems, Foster City, CA, USA) using MonAMP™ SYBR Green qPCR Mix (Monad, MQ10201, Wuhan, China). Each reaction consisted of 5 μL of SYBR qPCR master mix, 0.2 μM of forward and reverse primers, and 200 ng of cDNA in a final volume of 10 μL.

The cycling conditions were as follows: 95°C for 5 min, followed by 40 cycles of 95°C for 15 s and 60°C for 30 s. *PpTEF2* (Translation elongation factor 2) was used as the normalizing internal control. Relative gene expression levels were quantified according to the 2^–ΔΔCT^ method (Livak and Schmittgen, 2001). Three independent replicates were used for the experiments. The gene-specific primers for qPCR analysis are listed in **Supplementary Table S4**.

### 2.5 Sugars-target metabolome profiling

Sugar content was measured according to the previous protocol described by (Umer et al., 2020) with minor modifications. The same fresh shoot samples used for transcriptome analysis were employed for metabolome analysis. Each sample was ground with liquid nitrogen, and 0.02 g of tissue powder was vortexed with 0.5 mL extraction agent (methanol-isopropanol-water, 3: 3: 2; v/v/v) for 3 min. The mixture was then subjected to ultrasound in an ice water bath for 30 min.

Following centrifugation at 14000 r/min for 3 min, 50 μL of the supernatants was transferred to a 2 mL tube and mixed with 20 μL of internal standard (0.1 mg/mL ribitol). The mixtures were dried in a Speed Vac (Labconco, Kansas City, MO, USA), and then vortexed with 100 μL of methoxyamine hydrochloride (15 mg/mL). The samples were incubated at 37°C for 2 hours, followed by the addition of 100 μL BSTFA to each tube. The tubes were then incubated at 37°C for 30 minutes to obtain the derivative solution. The GC-MS assay was performed using a gas chromatograph coupled to a mass spectrometer (Agilent 8890-5977B, CA, USA) to quantify the sugar contents.

### 2.6 Weighted gene co-expression network analysis

The WGCNA package (v4.0.2) was used to assess the similarities of expression patterns among the samples based on transcriptome data. During data preprocessing, expression values with a mean less than 1 and a coefficient of variation less than 0.1 were excluded to minimize noise and focus on robust signals. In the networkType identification process, the network type was specified as ‘unsigned’, and the soft threshold power (β value) was determined automatically to optimize the network construction. The minimum module size was set to 30, and the minimum kME to stay was set to 0.3 to ensure robust module detection. The network of these relationships was further elucidated using Cytoscape software (v3.9.1, https://cytoscape.org).

### 2.7 Statistical analysis

All experiments were repeated at least three times for each study to ensure reliability and accuracy. Statistical differences were calculated using analysis of variance (ANOVA) in SPSS13.0 (IBM, USA), followed by a *t*-test to determine significant differences between means. The significance levels were set at * P < 0.05, ** P < 0.01, and *** P < 0.001.

## 3 Results

### 3.1 *N. parvum* inoculation induces peach gummosis

**Figure 1:**
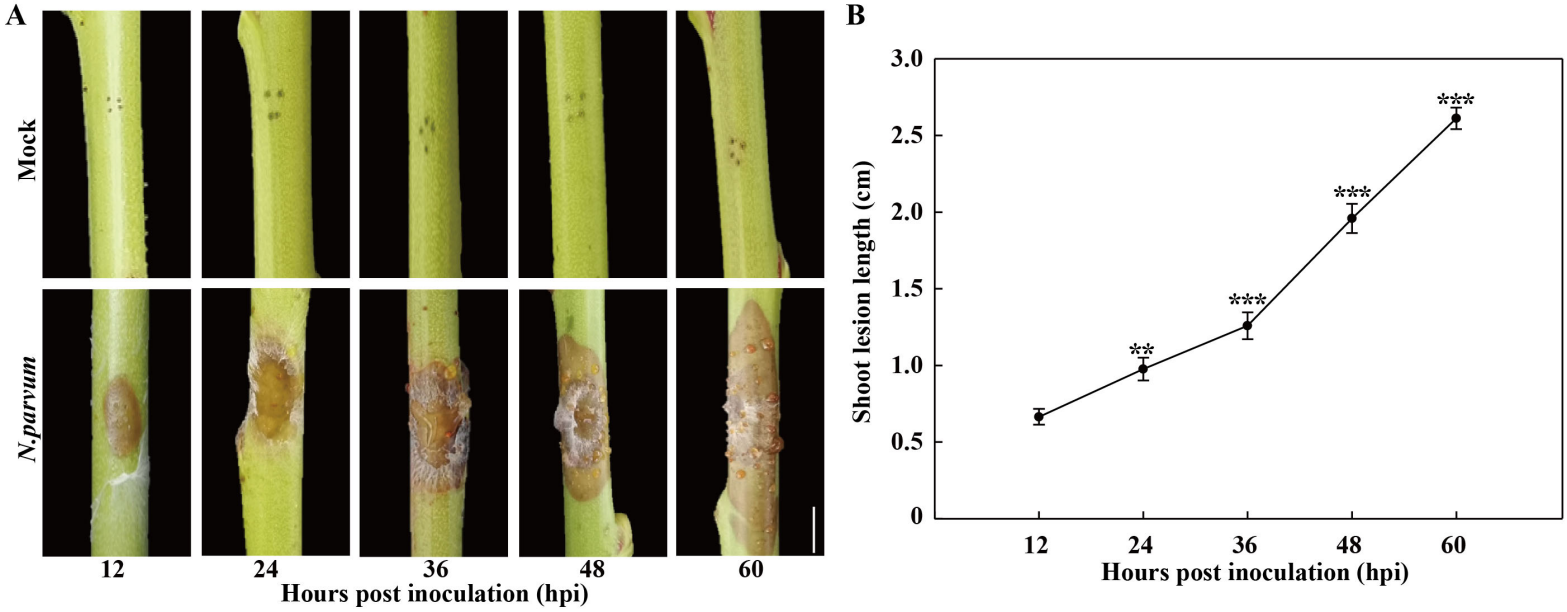
Development of peach gummosis in current-year shoots inoculated with *N. parvum* strain JSZ01. **(A)** Morphological progression of gummosis in current-year shoots wounded and inoculated without (mock) or with *N. Parvum*. **(B)** Lesion diameter in infected shoots at different time points. Bar in graph A is 0.5 cm. Error bars represent ± SD (n = 3). Asterisks indicate significant differences between 12 hours post inoculation (hpi) and the each other time points (**P < 0.01, ***P < 0.001).

The development of gummosis spots was observed on the shoots of ‘Spring snow’ peach trees with and without inoculation with *N. parvum* (**Figure 1A**). Compared to the mock group, the *N. parvum-*inoculated shoots exhibited visible necrotic lesion at 12 hours post inoculation (hpi), with a lesion length of approximately 0.7 cm. The lesions on peach shoots gradually expanded over time, reaching approximately 2.6 cm in length by 60 hpi (**Figure 1B**). Moreover, another typical symptom of gummosis was observed, as the lesions began releasing gum at 36 hpi (**Figure 1A**). In contrast, the mock treatment did not display any lesions or gum exudation. These results indicate that *N. parvum* inoculation induces peach gummosis.

### 3.2 Analysis of RNA-seq data

**Figure 2:**
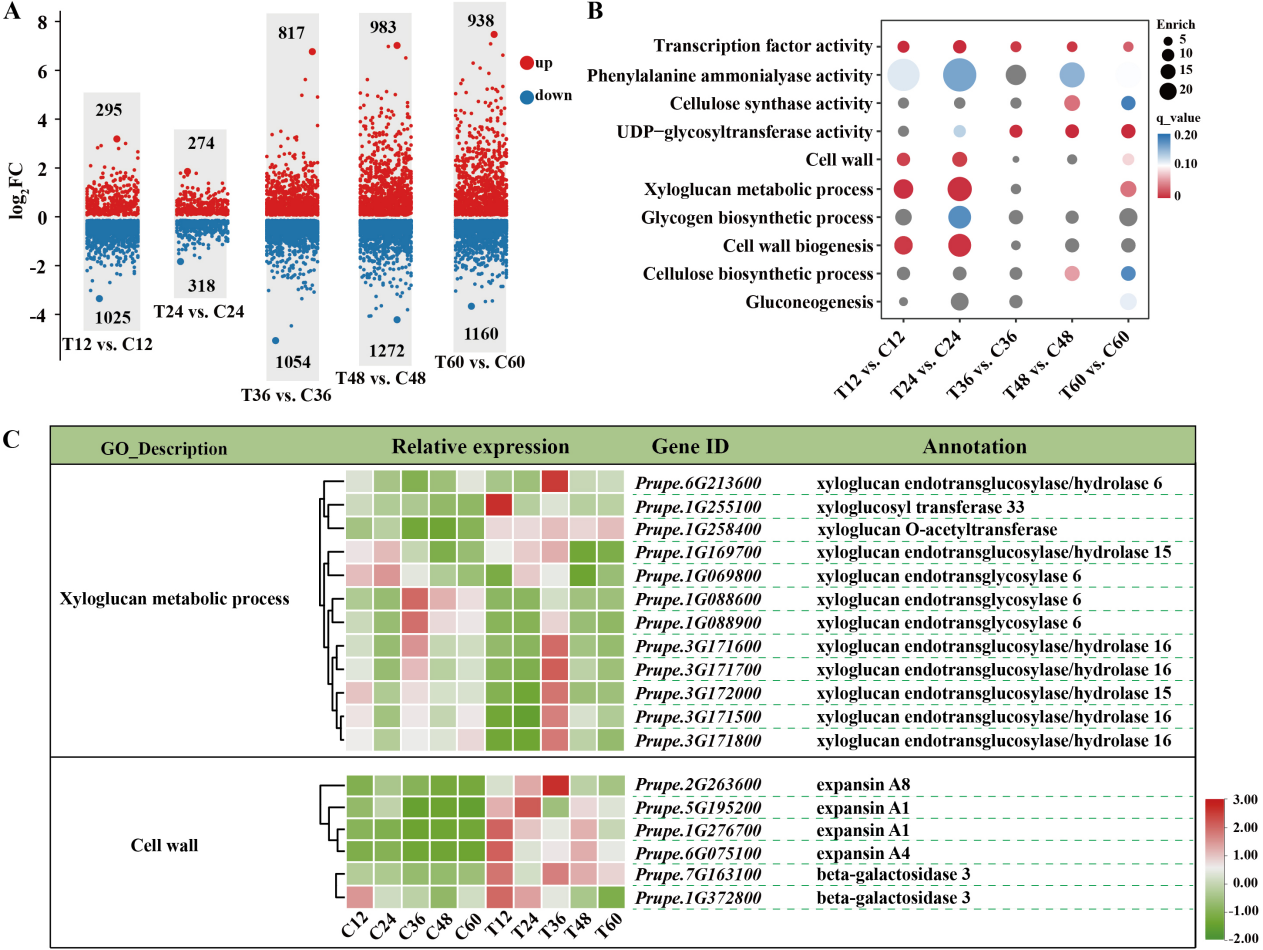
Transcriptomic analysis of peach shoots inoculated with Mock or *N. Parvum* at various time points. **(A)** Comparative analysis of differential expressed genes (DEGs) between mock **(C)** and *N. parvum*-inoculated (T) peach shoots at 12 h, 24 h, 36 h, 48 h, and 60 h. The numbers on the scatter diagram indicates the amount of DEGs. Up- and down-regulated DEGs are indicated with red and blue scatter, respectively. ‘T12 vs. C12’ indicates that the samples under *N. Parvum* infection treatment are compared with the mock at 12 h. **(B)** GO analysis of DEGs between different stages of mock and *N. Parvum* infection. The circle size indicted the DEGs count, and the circle color indicated q value. **(C)** DEGs involved in ‘xyloglucan metabolic process’ and ‘cell wall’ processes, indicating key genes related to cell wall modification and stress response.

RNA-seq was performed on samples from both the mock and *N. parvum* treatments at five time points. The number of total clean reads ranged from 38, 915, 380 to 58, 094, 244, with an average Q30 value of 95.42% and the GC content ranging from 45.43% to 46.34% (**Supplementary Table S1**). Principal component analysis (PCA) results (PC1 variance explained 25.27% and PC2 14.57%, R=0.1207) showed that each group sample has good repeatability, and the mock treatment groups have distinct global expression patterns (**Supplementary Figure S1**). A total of 8,136 differentially expressed genes (DEGs, log_2_fold≥1 with P-value≤ 0.05) were identified (**Supplementary Table S2**). Volcano plots showed upregulation of 295, 274, 817, 983, and 938 genes and downregulation of 1025, 318, 1054, 1272 and 1160 genes in comparisons between T12 vs. C12, T24 vs. C24, T36 vs. C36, T48 vs. C48, and T60 vs. C60, respectively (**Figure 2A**).

GO enrichment analyses revealed significant enrichment of DEGs in ‘xyloglucan metabolic process (GO:0010411)’, and ‘cell wall (GO:0005618)’ processes during the earlier periods of infection (12 hpi and 24hpi) (**Figure 2B**). Further analysis showed decreased expressions of *XTHs* (encoding for xyloglucan endotransglucosylase/hydrolases) and increased expressions of *EXPs* (encoding for expansin) and *BGALs* (encoding for beta-galactosidase) under *N. parvum* infection at 12 hpi and 24hpi (**Figure 2C**). At 12 hpi, the log_2_Fold change in the transcript level of *Prupe.1G088900* decreased by 2.6 times, while that of *Prupe.6G075100* increased by 2.58 times compared to the mock group (**Supplementary Table S2**). Moreover, DEGs were enriched in sugar metabolism-related processes, including ‘UDP-glycoyltransferase activity (GO:0008194)’, ‘gluconeogenesis (GO:0006094)’, and ‘Transcription factor activity (GO:0003700)’ process (**Figure 2B**). Collectively, these results demonstrated that cell wall metabolism, sugar metabolism, and transcription factors in peach shoots play important roles in responding to *N. parvum* infection.

### 3.3 Analysis of metabolites of peach shoots under *N. parvum infection*

**Figure 3:**
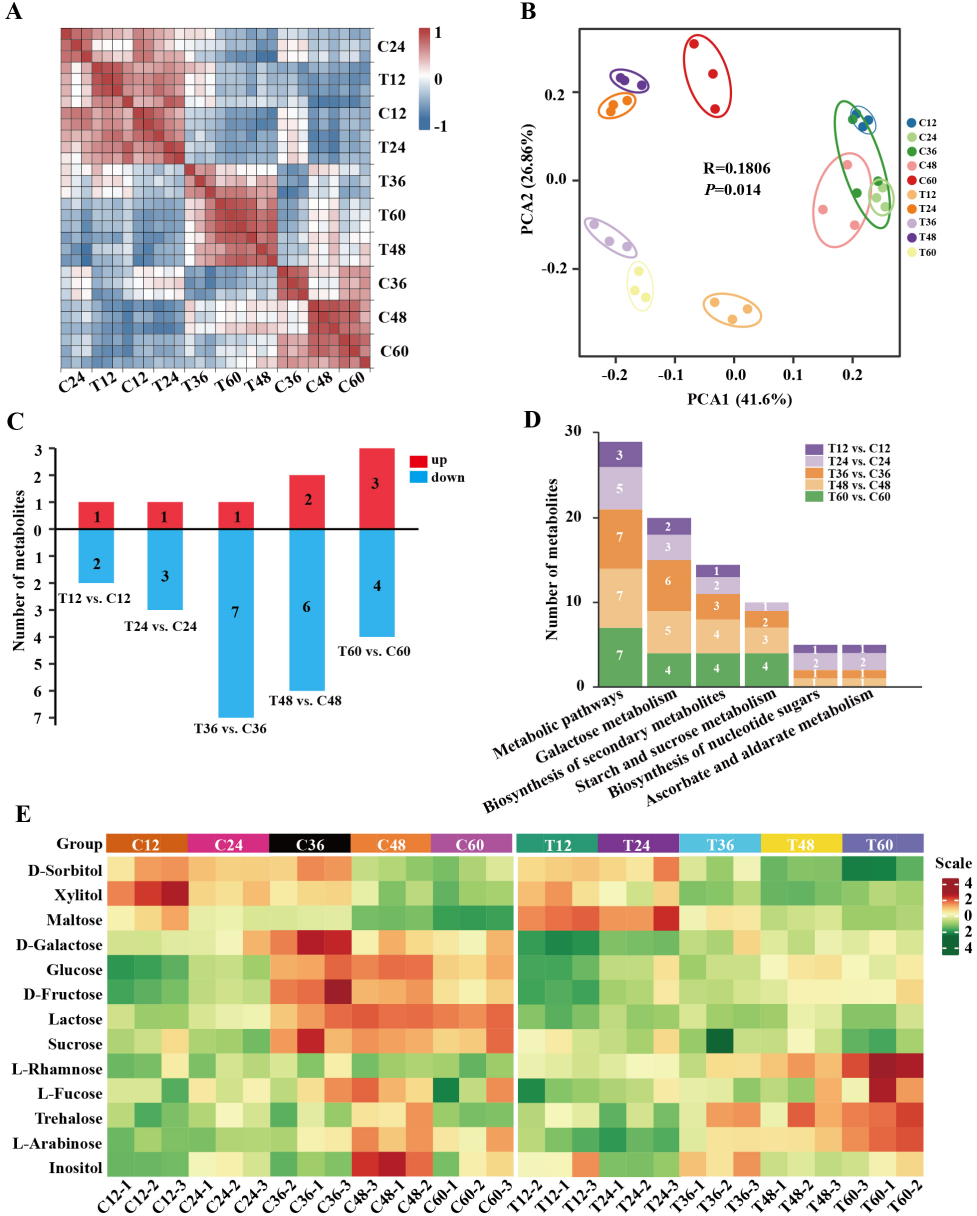
Metabolome analysis of peach shoots inoculated with Mock or *N. Parvum* at various time points. **(A)** Heatmap depicting correlation coefficient of metabolites of all samples. **(B)** PCA score plot of metabolite data. Each point represents an independent biological replicate. **(C)** Number of differential metabolites in metabolome at different time points. The numbers on the histograms indicates the amount of differential metabolites. Up- and down-regulated metabolites are indicated with red and blue histograms respectively. **(D)** KEGG pathway annotation for the metabolite profiles related to *N. Parvum* inoculation reflection. The numbers on the histograms indicates the amount of differential sugar metabolites at different time points. **(E)** Heatmap of metabolites. The heatmap shows the metabolite content of all samples, with low to high levels represented by a gradient of green to red.

Metabolome analysis was performed to investigate sugar levels in peach shoots during gum formation after *N. parvum* infection. A total of 13 sugar/alcohol metabolites were identified (**Supplementary Table S3**). The Pearson’s correlation matrix and PCA revealed good repeatability and distinct separation (PC1 variance explained 41.6% and PC2 26.86%, R=0.1806) of mock and *N. parvum* infection treatments (**Figures 3A, B**), indicating suitable for further analysis. Based on |Fold Change| >1 and *p-value* < 0.05, 13 sugar/alcohol metabolites showed differential accumulation: 3 (1 up-regulated, 2 down-regulated) in T12 vs. C12, 4 (1 up-regulated, 3 down-regulated) in T24 vs. C24, 8 (1 up-regulated, 7 down-regulated) in T36 vs. C36, 8 (2 up-regulated, 6 down-regulated) in T48 vs. C48 and 7 (3 up-regulated, 4 down-regulated) T60 vs. C60 comparisons (**Figure 3C**). KEGG pathway enrichment analysis of the differentially abundant sugar (DAS) showed that the DASs were mainly involved in ‘galactose metabolism’ pathway, ‘biosynthesis of secondary metabolite’ pathway, and ‘starch and sucrose metabolism’ pathway (**Figure 3D**). Compared to the mock group, the *N. parvum* infection group exhibited higher contents of maltose and L-rhamnose at 12-60 hpi, with maltose increasing 1.72-fold and L-rhamnose increasing 1.40-fold at 60 hpi. Trehalose contents elevated at 36-60 hpi, especially 1.86-fold increase at 60 hpi. In contrast, the content of D-galactose, fructose and glucose decreased at 36-60 hpi, with the most significant reduction at 36 hpi: 22% for D-galactose, 39% for fructose, 33% for glucose (**Figure 3E**; **Supplementary Table S3**). These results suggest that peach can regulate sugar metabolism pathways in response to *N. parvum* infection.

### 3.4 Correlation analysis of transcriptome and metabolome

**Figure 4:**
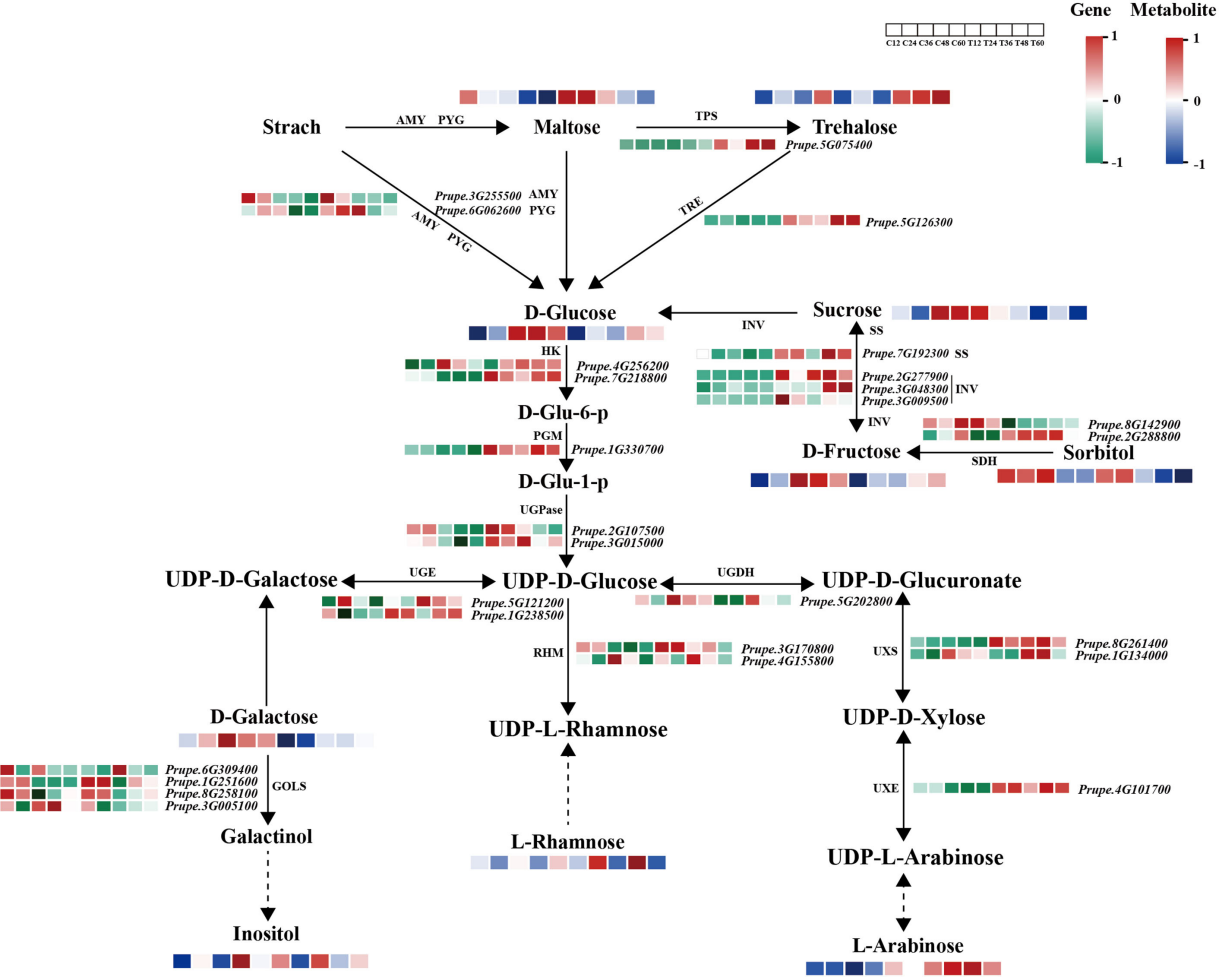
Expression profiles of structural genes and metabolites in the sugar metabolism pathway in mock and *N. parvum-*inoculated shoots. The heatmap shows the relative expression levels of structural genes on the basis of FPKM values, with low to high expression represented by a gradient of green to red. The metabolite levels are indicated by a blue and red color scheme, with blue representing low levels and red representing high levels.

To investigate the relationships between metabolites and genes in response to *N. parvum*-mediated peach gummosis, a sugar regulatory heatmap was generated to compare structural genes associated with sugar biosynthetic and decomposition pathways between *N. parvum*-infected and mock samples (**Figure 4**). This analysis revealed that transcripts of *AMY* and *PYG*, encoding α-amylase and α-Glucan phosphorylases (α-GPs), were upregulated by *N. parvum* infection. Additionally, genes involved in the synthesis or decomposition of maltose (*AMY*, *PYG*), trehalose (*TPS*: *trehalose-phosphate phosphatase*, *TRE*: *trehalase*), sucrose (*INV*: *invertase*, *SS*: *sucrose synthase*), sorbitol (*SDH*: *sorbitol dehydrogenase*), D-Galactose (*GOLS*: *galactinol synthase*) were differentially expressed under *N. parvum* infection. As UDP-sugar metabolism is a crucial step in polysaccharide formation, an in-depth analysis of the DEGs associated with UDP-sugar biosynthesis was performed. This revealed that transcripts of *HK* (coding for hexokinase), *PGM* (coding for phosphoglucomutase), *UGPase* (coding for UTP-glucose-1-phosphate uridylyltransferase), *UGE* (coding for UDP-glucose 4-epimerase), *UGDH* (coding for UDP-glucose 6-dehydrogenase), *UXS* (coding for UDP-glucuronate decarboxylase), *UXE* (coding for UDP-arabinose 4-epimerase), and *RHM* (coding for UDP-rhamnose synthase) genes were induced to varying degrees by *N. parvum* inoculation compared to the mock treatment.

### 3.5 Transcription factors respond to *N. parvum* infection

**Figure 5:**
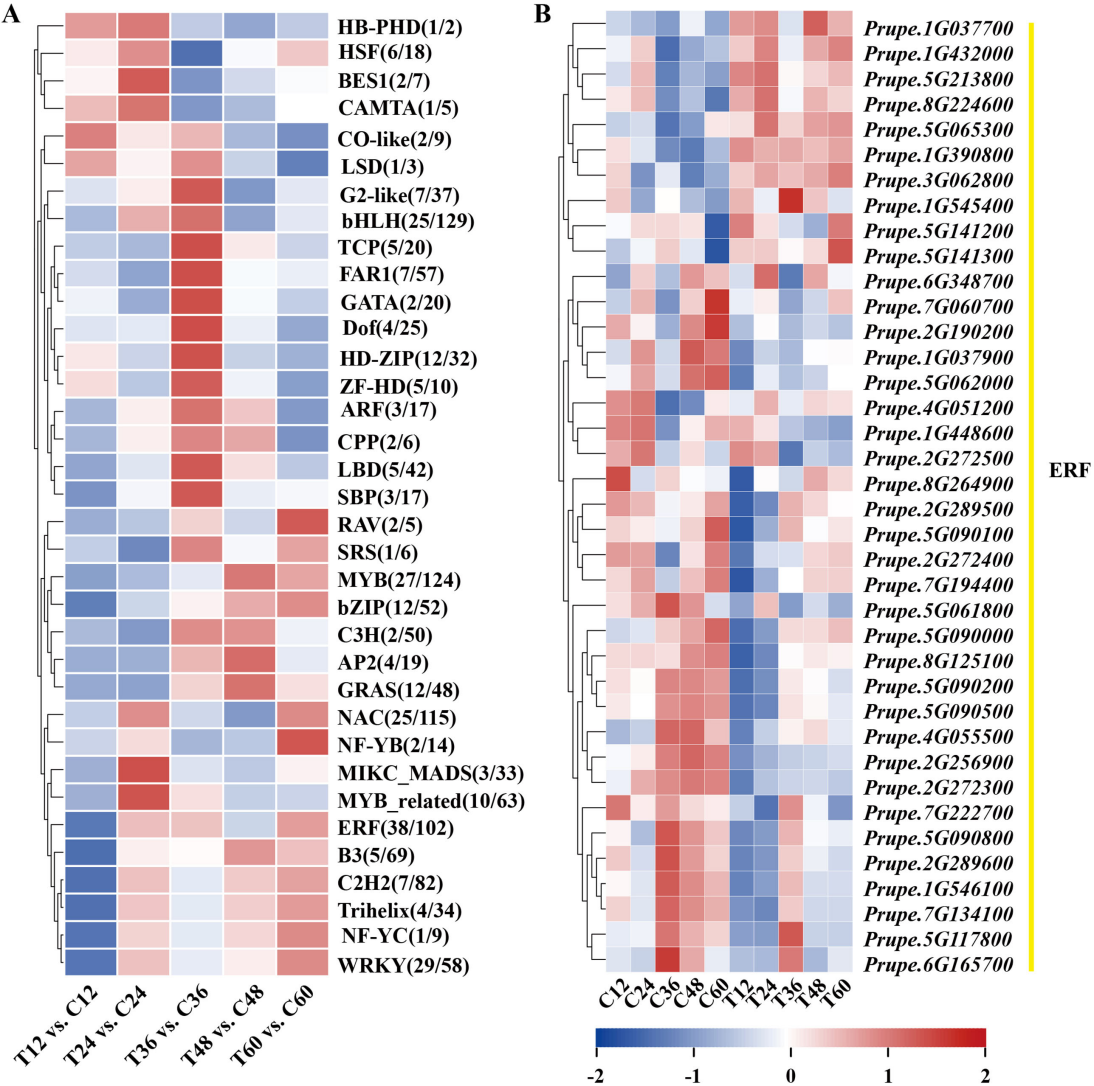
Expression dynamics of differentially expressed transcription factors families from the transcriptome profile. **(A)** Classification statistics of differentially expressed transcription factors families. **(B)** Heatmap of 38 differentially expressed ethylene-responsive factors (ERFs).

**Figure 6:**
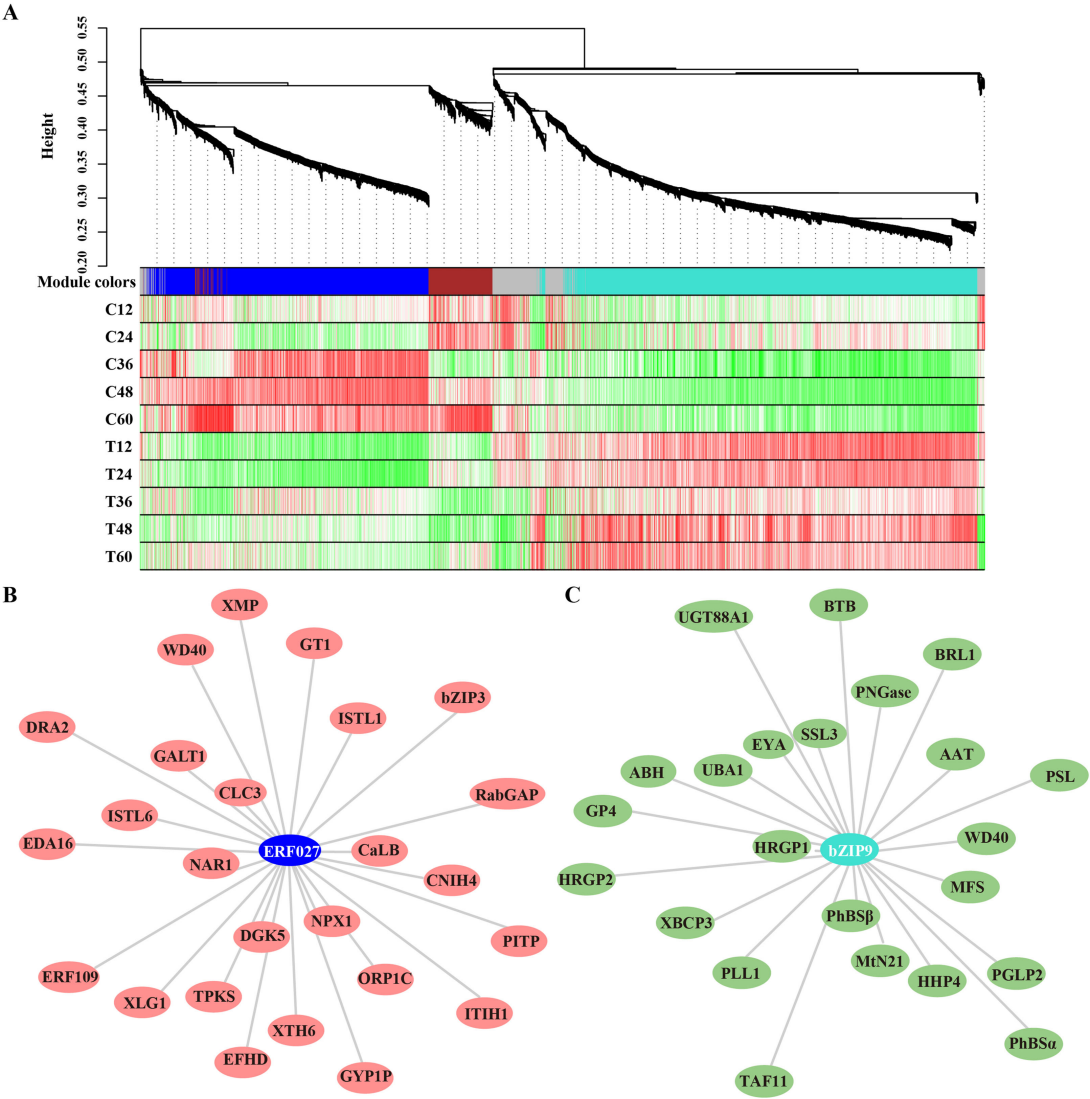
Gene modules identified by WGCNA assay based on the transcriptome profile. **(A)** Gene dendrogram and module colors were obtained by WGCNA. The major tree branches constitute 5 modules, marked with different colors. Heatmap depicting module–sample correlation. The correlation network analysis of ERF027 and bZIP9 and their major co-expressed genes from the blue **(B)** and turquoise **(C)** modules.

Transcription factors (TFs) play vital roles in plant disease response by directly targeting downstream genes. In this study, 277 TFs were differentially expressed in peach shoots inoculated with *N. parvum* at five time points compared to the mock group. These TFs were classified into 35 subfamilies based on the PlantTFDB databases. Notably, ERF TFs were the most numerous among these subfamilies. The heatmap analysis showed that 10 genes were significantly upregulated, while 28 genes were significantly downregulated in response to *N. parvum* infection (**Figures 5A, B**). In addition, WGCNA was performed to predict the key TFs and potential interacting genes. The identified genes were mainly concentrated in the blue, brown, grey, and turquoise modules (**Figure 6A**). *ERF027* and *bZIP9* were identified as the core TFs in the blue and turquoise modules, respectively (**Figure 6B, C**). Network analysis revealed different types of genes, including *galactosyltransferase 1* (*GALT1*), *xyloglucan endotransglycosylase 6* (*XTH6*), and *CaLB*, with high weight connected to *ERF027*, and *bZIP9* was highly correlated with genes such as *UDP-glucosyl transferase 88A1* (*UGT88A1*), *ubiquitin-activating enzyme 1* (*UBA1*) and *BRI1*. These findings indicate that *ERF027* and *bZIP9* may cooperate with their connected genes in response to *N. parvum* infection, highlighting the importance of TFs in *N. parvum*-mediated gummosis in peach, particularly *ERF027* and *bZIP9*.

### 3.6 Gene expression validation by qPCR

**Figure 7:**
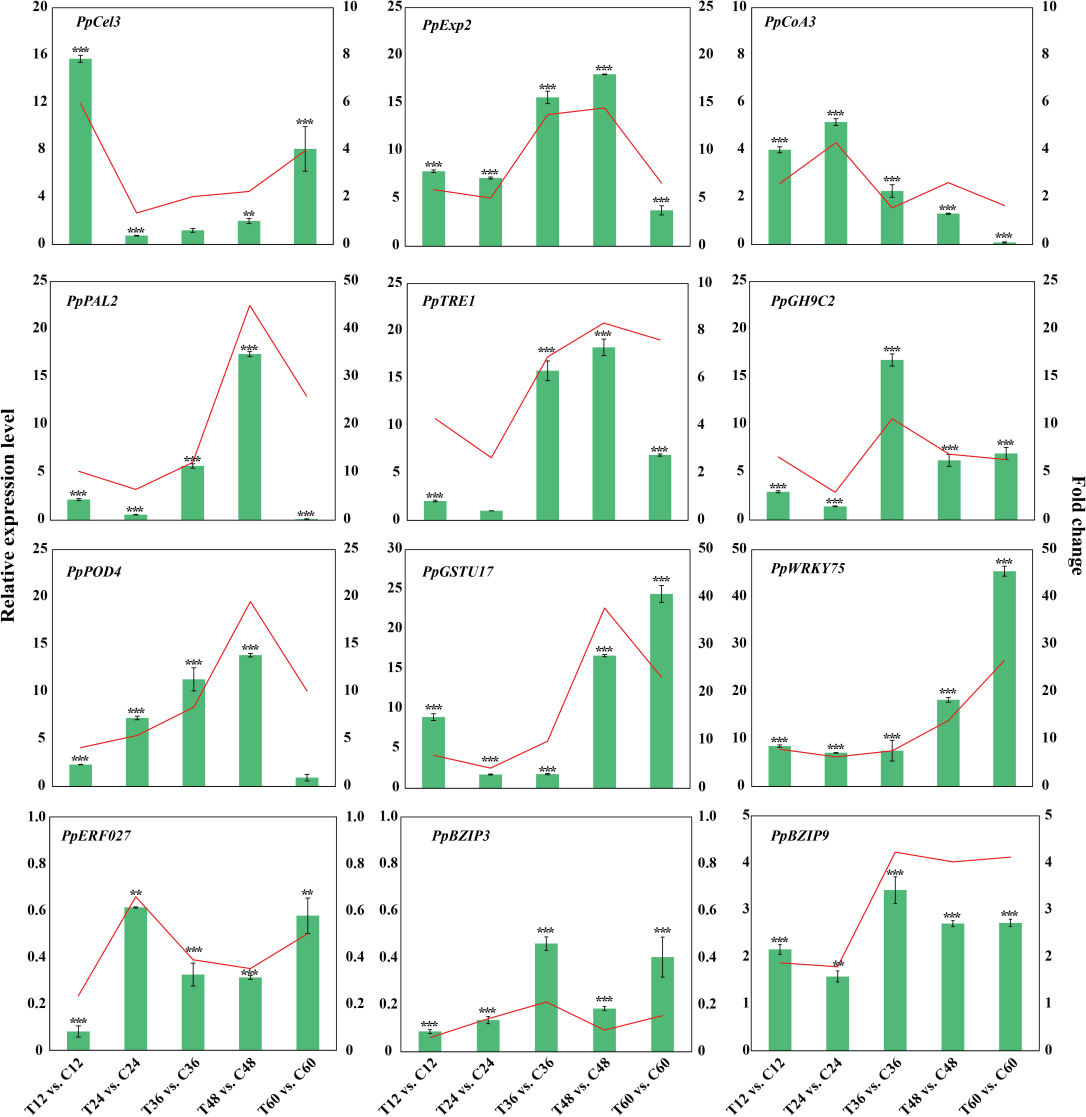
Validation of transcriptome data by qPCR for genes related to pathogen stress. The histograms and Line charts indicate the RNA-seq and qPCR results of 12 DEGs respectively.

To validate the gene expression results, 12 well-known pathogen stress-related genes involved in cell wall biogenesis (*PpCel3* and *PpExp2*), flavonoid biosynthesis (*PpCoA3* and *PpPAL2*), sugar metabolism (*PpTRE13* and *PpGH9C2*), ROS scavenging process (*PpPOD4* and *PpGSTU17*) and four TF (*PpWRKY75*, *PpERF027*, *PpbZIP3* and *PpbZIP9*) of peach were evaluated using qPCR (**Figure 7**). The correlation analysis of gene log_2_Fold change between qRT-PCR and RNA-seq data yielded a determination coefficient (R^2^) of 0.8385 (**Supplementary Figure S2**). These genes were induced to different extent by *N. parvum* infection, indicating that peach regulates a large number of stress-responsive genes to orchestrate its response to *N. parvum* infection.

## 4 Discussion

In this study, we examined both the symptom and molecular responses of peach shoots infected by *N. parvum*. Our results revealed that *N. parvum* infection leads to the formation of necrotic lesions and gum exudation on peach shoots, which mirrors the symptoms observed in shoots inoculated with *L. theobromae* (Gao et al., 2016). This suggests that *N. parvum* (JSZ01) exhibits strong pathogenicity in peach trees.

The cell wall plays a crucial role as a physical barrier in responding to pathogen attacks, and its integrity affects a plant’s susceptibility to pathogens (Xu et al., 2022; Xiao et al., 2024). Our transcriptomic analysis showed that *N. parvum* infection altered the transcripts of *EXPs* and *BGALs* involved in cell wall processes. Expansin (encoded by *EXPs*) and β-galactosidase (encoded by *BGALs*) are proteins that loosen the plant cell wall framework (Lv et al., 2020; Molina et al., 2024; Su et al., 2024). The induced transcripts of *EXPs* and *BGALs* suggest that *N. parvum* infection causes plant cell wall breakdown. Furthermore, our results showed that DEGs involved in xyloglucan metabolism, particularly *XTHs*, were expressed to different degrees under *N. parvum* infection. Xyloglucans, a type of hemicellulose, form a network with cellulose microfibers to maintain cell wall tensile strength. XTH enzymes encoded by numerous *XTH* genes have dual functions: xyloglucan endotransglucosylase (XET) activity and/or xyloglucan endohydrolase (XEH), which can reconnect or cleave xyloglucan chains (Yan et al., 2019; Nishikubo et al., 2011). Previous research has shown that pathogenic fungi produce enzymes that inhibit *XTHs* expression and reduce XET enzyme activity to infect host plants. The suppressed transcripts of most *XTHs* in the early stage of *N. parvum* infection support this theory. Therefore, changes in xyloglucan metabolism and cell wall processes are essential to *N. parvum* to successfully infect peach shoots.

The gum effluxion observed after *N. parvum* infection has been reported to mainly consist of polysaccharides (Simas et al., 2008; Decker and Kleczkowski, 2019), but the associated sugar metabolism remains unclear. Our sugar metabolites analysis revealed that, at the early stage (12 or 24 hpi), the contents of maltose and sucrose were higher in the *N. parvum* infection group than in the mock group. In the same samples, transcripts of *AMY*, *PYG*, *INV* and *SS* genes were upregulated. These sugars have been reported to act as molecular messengers or osmotic protective substances, regulating various physiological responses to adverse environmental stresses (Morkunas and Ratajczak, 2014; Dahro et al., 2016; Singh et al., 2015). At the early stage of *N. parvum* infection, peach may promote the accumulation of maltose and sucrose by upregulation gene related to transport and synthesis to protect themselves. At the gum overflow stage (36 to 60 hpi), GO analysis showed that DEGs associated with the ‘UDP-glycosyltransferase activity’ pathway were enriched. Additionally, the transcript levels of genes encoding key enzymes involved in UDP-sugar synthesis, such as *UGPases*, *RHMs*, and *UXSs*, were significantly promoted compared to the mock group. Correspondingly, the concentrations of sugars which could be decomposed or converted into UDP sugars, such as D-galactose, D-Glucose, D-Fructose, were decreased under *N. parvum* stress. Combined with the decrease of glucose and fructose contents under *L. theobromae* infection (Li et al., 2014b), we suggest that UDP sugar metabolism is the primary factor in gum formation. Genes involved in sugar biosynthesis and degradation may play a crucial role in responding to *N. parvum* stress. However, further in-depth research is needed to clarify these mechanisms.

The crucial roles of transcription factors (TFs) in plant immune responses have been well documented (Jiang et al., 2021; Song et al., 2022; Li et al., 2024). In this study, we identified 277 DEGs as TFs, with the ERF family being the most prominent. As ethylene responsive factors, *ERFs* are involved in multiple plant pathogen stress responses (Nie and Wang, 2023; Ma et al., 2024). For example, *VaERF16* enhances grapevine resistance to *Botrytis cinerea* infection by upregulating defense genes related to the JA/ET signaling pathway (Zhu et al., 2022). *Magnaporthe grisea* infection repressed the expression of some *ERFs* in rice, especially *OsERF027* (Pegoraro et al., 2013). Previous studies have shown that exogenous ethylene treatment increases the extent of gummosis caused by wound or pathogen infection (Li et al., 2014b; Gao et al., 2022; Zhang et al., 2022). In gummosis-resistant variety ‘Da Hongpao’ and gummosis-susceptible variety ‘Spring snow’, *PpERF1-1/2* and *PpERF98-1/2* are induced by *L. theobromae* and form a transcriptional cascade to negatively regulate the response to *L. theobromae* infection (Zhang et al., 2023). Our RNA-seq data revealed that the expression of *PpERF1-1* and *PpERF98-1/2* was slightly induced by *N. parvum* infection. In contrast, *PpERF1-2* expression was rapidly and continuously upregulated by *N. parvum* infection, compared to the mock group (**Supplementary Figure S3**). Additionally, *ERF027* was identified as a central TF in the down-regulation network involving various genes. Our findings highlight the critical role of ERF-mediated ethylene signaling pathway in regulating the peach gummosis response. Moreover, a basic leucine zipper family member, *bZIP9*, was identified as a key TF in the up-regulation network. Emerging evidence indicates that bZIP proteins are involved in plant responses to various pathogen infections. For instance, *AtbZIP59* positively regulates resistance to *Pst* DC3000 by controlling stomatal immunity (Song et al., 2022), and *RhbZIP17* are positive regulators of lignin accumulation by upregulating *RhCAD1* genes to increase rose tolerance to *Botrytis cinerea* (Li et al., 2024). In this study, we found that *bZIP9* exhibited a strong correlation with various genes, including *BRI1*, *UGT88A1*, *and UBA1*. Previous studies have shown that *bZIP9* plays a role in seed germination, as well as in salt, and drought stress tolerance (Ortiz-Espín et al., 2017; Zhu et al., 2021; Diallo et al., 2024). Our findings suggest that *bZIP9* may interact with these genes to respond to peach gummosis through the brassinosteroid biosynthesis pathway, UDP-sugar metabolism, and the ubiquitination pathway. Therefore, we propose that *ERF027*, *bZIP9*, and other stress-related genes play a crucial role in regulating the response to *N. parvum* infection.

## Data Availability

The datasets presented in this study can be found in online repositories. The names of the repository/repositories and accession number(s) can be found below: PRJNA1149560 (SRA).
