# Supplementary material for: Transcriptomic and sugar metabolic analysis reveals molecular mechanisms of peach gummosis in response to Neofusicoccum parvum infection
Source: Front Plant Sci. 2024 Oct 11;15:1478055. doi: 10.3389/fpls.2024.1478055 (PMC11503026; doi:10.3389/fpls.2024.1478055)
Supplement: Supplementary file 1 [file DataSheet1.zip › Data Sheet 1/Table 1.DOCX]

**Transcriptomic and sugar metabolic analysis reveals molecular mechanisms of peach gummosis in response to *Neofusicoccum parvum* infection**

**Supplementary Materials:**

**Figure S1.** PCA score plots of gene expression. Each point represents an independent biological replicate.

**Figure S2.** Comparison between the log_2_Fold change of gene expression ratios obtained from RNA-seq and qRT-PCR data. The determination coefficient (R^2^) between qPCR and RNA-seq is shown as R^2^ = 0.8385.

**Figure S3.** Heatmap of *ERF1-1/2* and *ERF98-1/2*.

**Table S1.** RNA sequencing data analysis summary.

**Table S2.** Summary of DEG genes identified by gene annotation of RNA sequencing data.

**Table S3.** The 13 metabolites content detected by sugar metabolomics in Mock and *N. Parvum* infection groups at different stages.

**Table S4.** Primers used in this study.


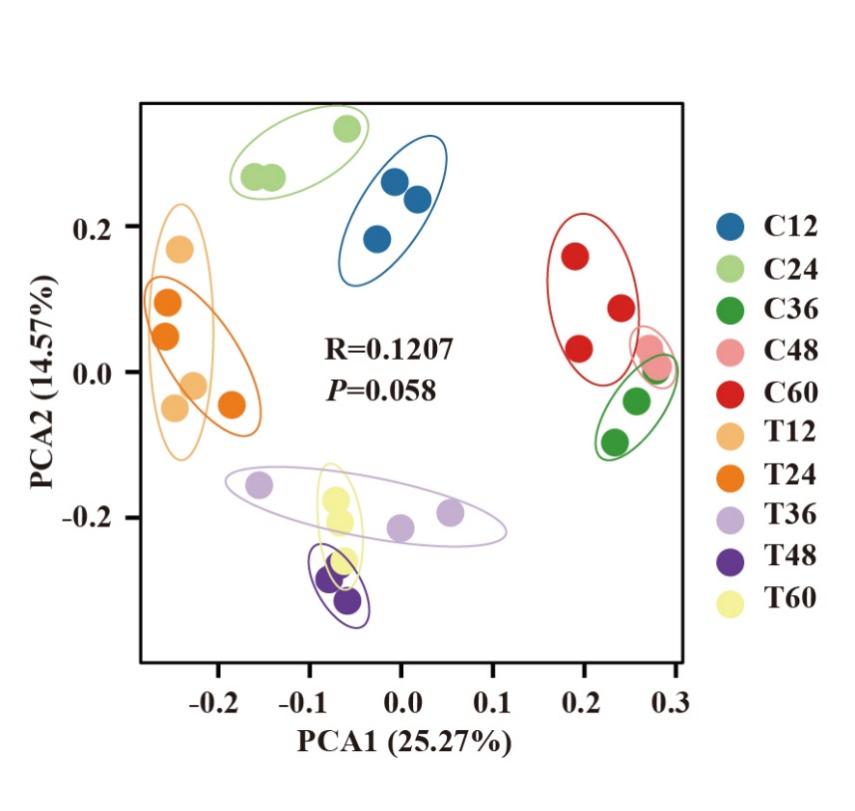


**Figure S1.** PCA score plots of gene expression. Each point represents an independent biological replicate.

**
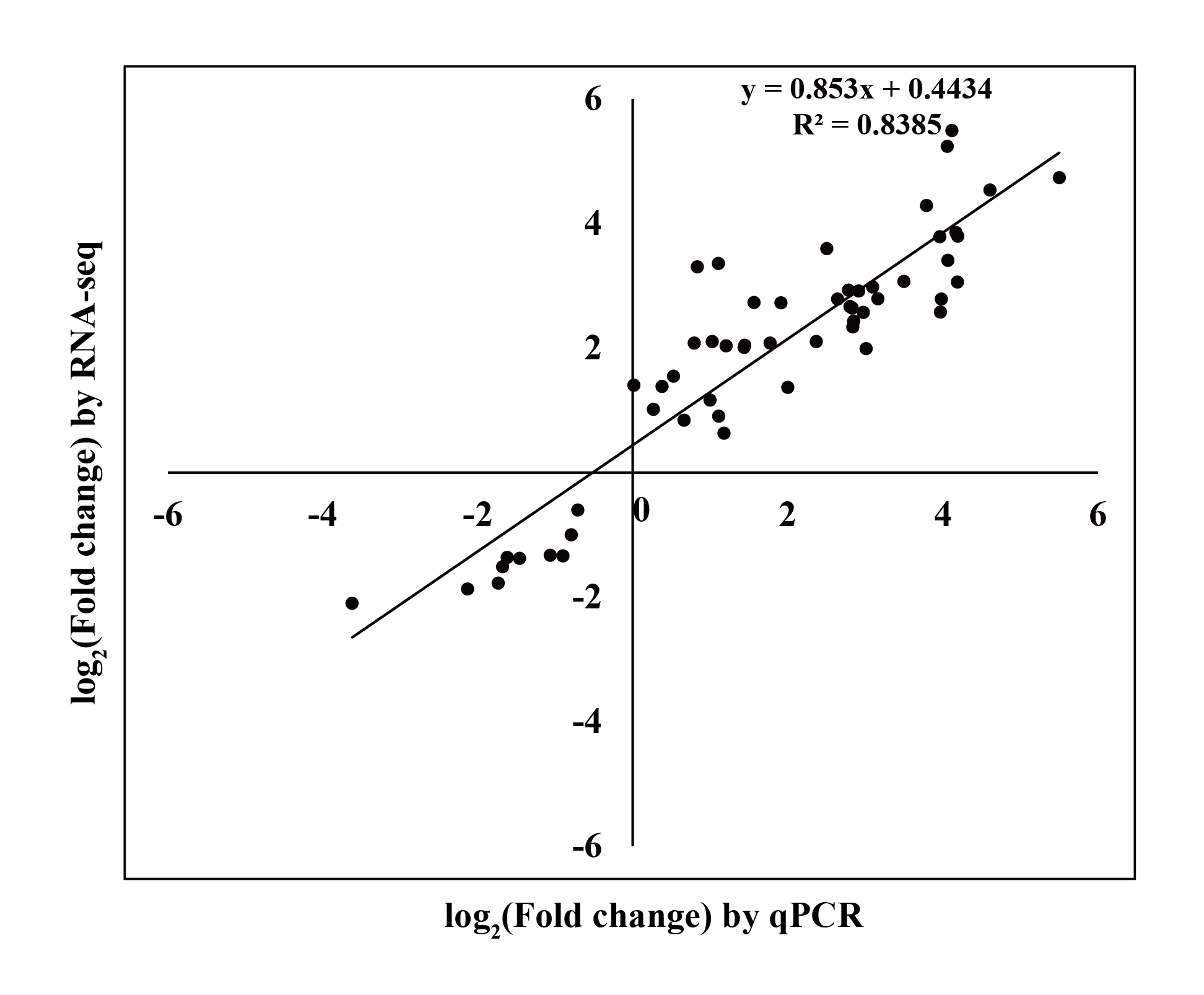
**

**Figure S2.** Comparison between the log_2_Fold change of gene expression ratios obtained from RNA-seq and qRT-PCR data. The determination coefficient (R^2^) between qPCR and RNA-seq is shown as R^2^ = 0.8385.

**
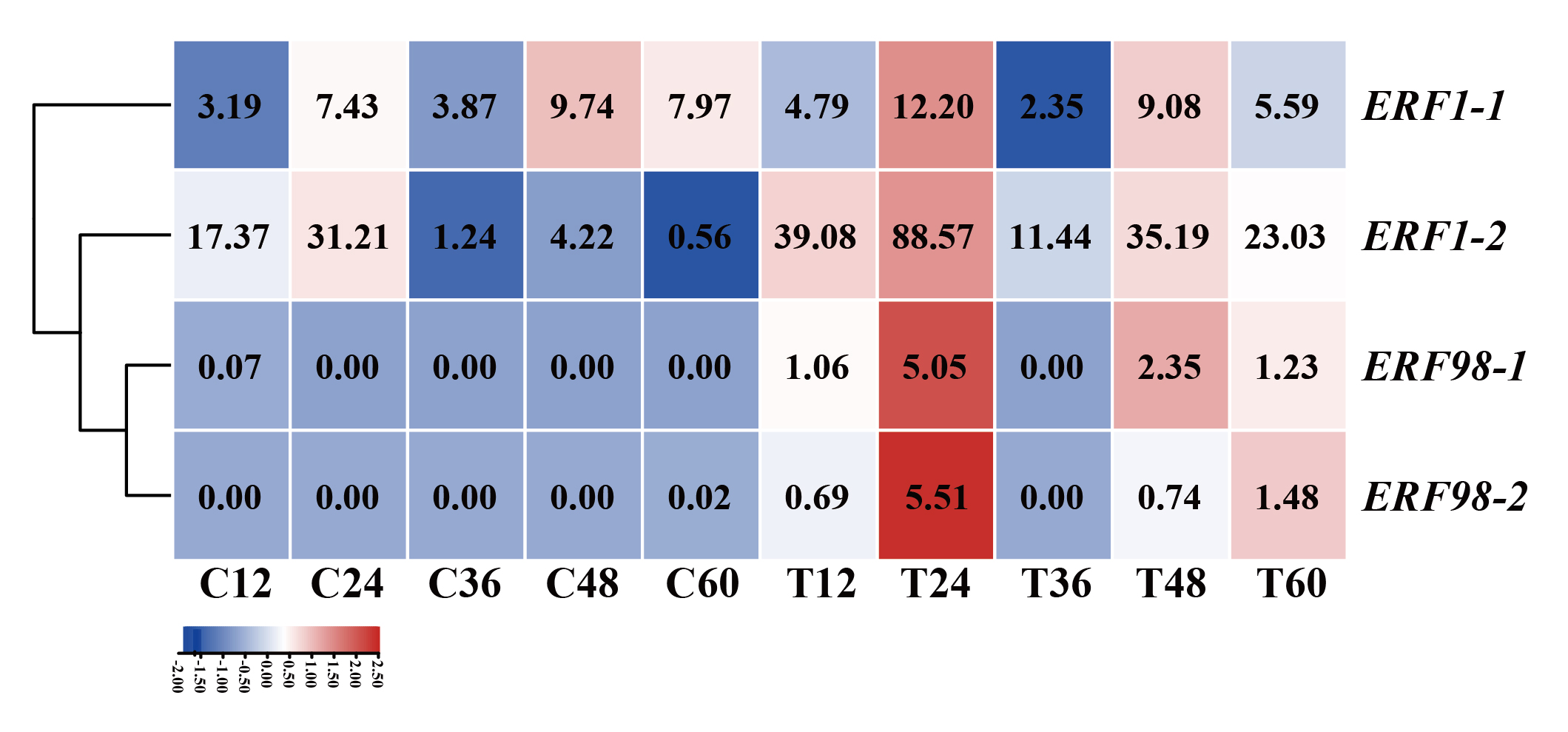
**

**Figure S3.** Heatmap of *ERF1-1/2* and *ERF98-1/2*.

**Table S1 RNA sequencing data analysis summary**

| **Sample** | **Total Clean Reads** | **GC(%)** | **Q20(%)** | **Q30(%)** | **Uniquely mapping gene ratio (%)** |
| --- | --- | --- | --- | --- | --- |
| C12-1 | 41124004 | 45.75 | 99.28 | 95.35 | 96.43 |
| C12-2 | 50001900 | 45.77 | 99.36 | 95.81 | 96.33 |
| C12-3 | 54188714 | 45.76 | 99.29 | 95.42 | 96.22 |
| C24-1 | 50544104 | 45.72 | 99.29 | 95.36 | 96.14 |
| C24-2 | 47152416 | 45.65 | 99.26 | 95.15 | 96.33 |
| C24-3 | 47510848 | 45.66 | 99.35 | 95.79 | 96.25 |
| C36-1 | 49671074 | 45.84 | 99.21 | 94.89 | 96.36 |
| C36-2 | 47704000 | 45.9 | 99.28 | 95.32 | 95.81 |
| C36-3 | 47559028 | 45.92 | 99.25 | 95.14 | 96.6 |
| C48-1 | 45821870 | 45.99 | 99.28 | 95.38 | 95.71 |
| C48-2 | 40588648 | 46.34 | 99.31 | 95.56 | 93.98 |
| C48-3 | 49551830 | 45.94 | 99.26 | 95.24 | 95.26 |
| C60-1 | 52924944 | 45.88 | 99.32 | 95.59 | 96.13 |
| C60-2 | 47107168 | 45.83 | 99.23 | 95.03 | 96.36 |
| C60-3 | 58094244 | 45.63 | 99.4 | 95.78 | 94.00 |
| T12-1 | 46747408 | 45.61 | 99.33 | 95.6 | 96.14 |
| T12-2 | 47083780 | 45.6 | 99.32 | 95.54 | 96.21 |
| T12-3 | 45205076 | 45.55 | 99.33 | 95.6 | 96.12 |
| T24-1 | 51601102 | 45.43 | 99.34 | 95.63 | 96.04 |
| T24-2 | 57312916 | 45.57 | 99.24 | 95.08 | 95.83 |
| T24-3 | 50436762 | 45.46 | 99.32 | 95.57 | 96.10 |
| T36-1 | 56149318 | 45.72 | 99.31 | 95.5 | 96.25 |
| T36-2 | 45263626 | 45.68 | 99.32 | 95.6 | 96.10 |
| T36-3 | 38915380 | 45.78 | 99.29 | 95.4 | 96.47 |
| T48-1 | 52811456 | 45.73 | 99.36 | 95.81 | 95.80 |
| T48-2 | 40369514 | 45.7 | 99.27 | 95.27 | 95.89 |
| T48-3 | 46475002 | 45.72 | 99.28 | 95.3 | 95.69 |
| T60-1 | 49140800 | 45.61 | 99.28 | 95.3 | 96.10 |
| T60-2 | 54024302 | 45.81 | 99.27 | 95.24 | 95.23 |
| T60-3 | 49122690 | 45.63 | 99.28 | 95.32 | 95.56 |

Note: “C”: mock-inoculated peach shoots, “T”: inoculated peach shoots.

**Table S4** **Primers used in this study**

| **Gene Name** | **Forward primer (5’-3’)** | **Tm** | **Reverse primer (5’-3’)** | **Tm** | **Product size size size** |
| --- | --- | --- | --- | --- | --- |
| *PpTEF2* | AGCAAGTCACCCAACAAGCATA | 57.4 | CCAACCAAACTCTTCAGCCAAT | 56.6 | 147 |
| *PpCel3* | GCGGCAGCCTCATTGTCACTCCG | 65.1 | GGACCTCCCACAATGGCTCCAACC | 64.6 | 265 |
| *PpExp2* | CTGCAGCACTGAGCACTGC | 60.2 | GCTGGAAGACAGGCTGAGAGAGG | 61.7 | 216 |
| *PpCoA3* | GACGACGTGGTACTGTGCG | 58.9 | CGAGCTCAGGTCGAACTGC | 58.9 | 228 |
| *PpPAL2* | ATGGCAACCAACTCCATCAAGC | 58.9 | GTCCATGACCCAGTCGCTGC | 61.5 | 300 |
| *PpTRE1* | CGCAGAGAAGGGACAATGGC | 59.4 | CATGTGTTGGATTGGAGCCCAGCC | 63.3 | 280 |
| *PpGH9C2* | GGTGACTCCATGGGTGGAACTGG | 62.4 | CTGTGGCCAAGAAGGAGGCAC | 61.5 | 277 |
| *PpPOD4* | GCCATTGCTGCTCGAGACTC | 59.3 | CTCGTTGTATATGCGAGCTCTG | 55.9 | 258 |
| *PpGSTU17* | TGATCGCGCCACGGCACG | 64.2 | CTCTATGACTCTGAGCCACC | 55.1 | 250 |
| *PpWRKY75* | CATCAGCAGGAAACCACG | 54.9 | CGGTAGCCATCGTCAAG | 53.8 | 256 |
| *PpERF027* | ACCGCGGCATCCGGTGCC | 66.9 | CAGAGCGAGTGCGGCCACA | 63.2 | 137 |
| *PpERF109* | ATGCCCTTGCACGCCAATCGC | 64.2 | GGTCCGTTGCTGATTGGGTC | 59.4 | 199 |
| *PpbZIP9* | CCAGCATCTCAGCAACCATGG | 59.5 | CGCAGACTCCCTATTCGAGACC | 59.5 | 230 |
